# The Influence of Juvenile Graves' Ophthalmopathy on Graves' Disease Course

**DOI:** 10.1155/2017/4853905

**Published:** 2017-10-31

**Authors:** Jurate Jankauskiene, Dalia Jarusaitiene

**Affiliations:** Eye Clinic of Lithuanian University of Health Sciences, Kaunas, Lithuania

## Abstract

**Purpose:**

To investigate juvenile Graves' ophthalmopathy (GO) signs and compare Graves' disease (GD) course in patients with or without GO.

**Patients and Methods:**

There were analyzed data (visual acuity, proptosis, palpebral fissure measurements, clinical activity score (CAS), and the course of GD) of 67 children who have been newly diagnosed with GD. 26.9% of patients with GD had signs of ophthalmopathy (GO+), and 73.1% were without ophthalmopathy (GO−).

**Results:**

Upper eyelid retraction (72.3%), proptosis (66.7%), and soft tissue changes (27.8−38.9%) were in GO+ patients. The palpebral fissure, CAS, and proptosis values were greater in the GO+ group than in the GO− group (*p* < 0.001). GD course in GO+ patients was longer than that in GO− patients (*p* < 0.001). The duration of the first remission was longer in GO− than in GO+ patients (*p* < 0.001). The duration of first remission was longer than one year for 61.2% in GO− and 33.3% in GO+ patients (*p* < 0.02).

**Conclusion:**

The common manifestations of juvenile GO patients were upper eyelid retraction, proptosis, and soft tissue involvement. The study demonstrates that pediatric patients with GO are more likely to have a severe course of autoimmune thyroid disease.

## 1. Introduction

Juvenile GD is a rare disease; thus, its clinical signs are less well defined in comparison with adults [1–4]. The frequency of GO in children is low, about 1.7 to 3.5 cases per 100.000 population per year [1, 5]. Juvenile GO usually occurs during the hyperthyroid state, and the clinical manifestation is mild and self-limited [1, 5–7]. Severe ophthalmopathy in pediatric age is uncommon [1, 8]. According to low incidence of GO between children, manifestation of this disease is poorly analyzed and mainly investigated in adults. This raises the risk of the identification of GO signs too late. Adult patients with GD treated with antithyroid drugs achieve long-lasting remission from 40 to 60% [9, 10]. There have been few studies of the relationship between the duration of therapy with antithyroid drugs and the relapse risk in pediatric patients. Studies demonstrated that in children with GD the treatment with antithyroid drugs is offered about two years and this time could be considered reasonable before deciding to choose other methods of the treatment [3, 8, 11]. Asian ethnic children patients have a higher rate of remission (46.1–56.6%) with antithyroid therapy [2, 12, 13]. Other authors found that remission was achieved in 12–33% of children with GD [14–16]. Authors reported that 44% of adult GD patients relapsed and remission occurred in 57% of patients [17]. Liu et al. [9] showed that ocular signs in GD influence the severity of this disease in adults. Often, relapses of hyperthyroidism are accompanied by the worsening of coexisting GO. Numerous controlled prospective and retrospective studies have investigated the impact of many predictive factors in adult GD course; however, the influence of GO on the course of GD in children has not been studied. Few studies showed the impact of GO on remission or relapse rates of adult Graves' hyperthyroidism [18–20]. The treatment and long-term remission or relapse rates following antithyroid drug therapy in juvenile patients with GD, and ophthalmopathy results are controversial and remain debatable. Authors show that patients with GD who relapsed or remained in remission did not differ with respect to age, goiter size, GO, and serum T4 or T3 [21]. Quadbeck et al. [10] and Mohlin et al. [17] showed that GO does not affect the course of GD. Therefore, the aim of this study was to investigate juvenile GO signs and compare GD course in patients with or without GO.

## 2. Patients and Methods

We analyzed data of medical records of 67 children who have been newly diagnosed with GD at an age below 18 years and treated at Kaunas Clinic of Lithuanian University of Health Sciences, during the period from January 2002 to August 2012. The present study protocol was approved by the Kaunas Regional Biomedical Research Ethics Committee. Written informed consent was obtained from the parents or guardians of the children who participated.

26.9% patients with GD had signs of ophthalmopathy (GO+), and 73.1% had none (GO−). The diagnosis of GD was made by an endocrinologist and based on commonly accepted clinical and laboratory criteria: clinical symptoms of hyperthyroidism, increased serum concentrations of free thyroxin (FT4), free triiodothyronine (FT3), suppressed thyrotropin (TSH) concentrations, increased serum concentrations of TSH receptor antibodies (TSHR Ab), and diffuse goiter on palpation and ultrasound. Clinical activity score based on seven signs of orbital inflammation was calculated for each patient at the initial visit [22]. GO severity was evaluated according to the European Groups on Graves' Orbitopathy (EUGOGO) recommendations [23]. We evaluated patient's palpebral aperture (measured in millimeters), soft tissue involvement, proptosis (measured in millimeters with the use of the Hertel exophthalmometer), extraocular motility, and visual acuity (Snellen chart).

We also evaluated data regarding the course of GD: the duration of the first treatment and remission, and rate of relapses within one and two years after remission.

### 2.1. Statistical Analysis

Statistical analysis was performed using SPSS software program package (version 20.0; SPSS Inc., Chicago, IL, USA). The following statistical characteristics were expressed as the mean value and standard deviation (SD). An independent sample *t*-test and the Mann–Whitney *U* test were used as well. Categorical variables were compared with the use of the chi-square test or Fisher's exact test. A *p* value of less than 0.05 was considered to be significant.

## 3. Results

The median age of all patients at Graves' disease diagnosis was 13.3 ± 2.3 (4.4–18) years. No statistical difference was found between GO+ and GO− regarding the age of GD onset. The age of GO− patients was 12.7 ± 2.9 (4.4–18) years. The age of GO+ patients was 13.9 ± 3.4 (5.8–18) years (*p* = 0.09) (Table 1).

GO has occurred together or to GD diagnosis in 83.3% of patients. Comparing the development of GO in GD patients by gender, we found that the female gender in both (GO+ and GO−) groups was statistically significantly more than males (83.3% females/16.7% males in the GO+ group, ratio 5 : 1, versus 81.6% females/18.4% males in the GO− group, ratio 4.4 : 1), but between the groups, it was not significant (*p* > 0.05). 58 patients with GD (86.6%) were nonsmokers; 9 patients (13.4%) aged 15–18 years were smokers. 14 patients (20.9%) had household members who smoked.

Of 67 patients with GD, 85.1% had been treated with thionamide antithyroid drugs, mostly with methimazole, others 9.0% underwent replacement therapy, and 5.9% underwent thyroidectomy. The course of GD in GO+ lasted 28.9 (from 13.5 to 87.0) months and in GO− 18.7 (from 13.5 to 51.4) months.

The results showed that best-corrected visual acuity in the GO+ group was 0.9 ± 0.1 versus 1.0 ± 0.1 in the GO− group (*p* > 0.05). The most frequent specific manifestations were upper eyelid retraction (72.3%), proptosis (66.7%), and Graefe's symptom (44.4%). The palpebral fissure (12.9 ± 1.3 mm) (range from 11.5 to 14 mm) was statistically significantly wider in GO+ than in GO− (11.3 ± 0.6 mm) (from 11 to 13 mm) (*p* < 0.001). Proptosis (17.4 ± 1.7 mm) (range 16.5–19 mm) in GO+ patients was greater than that in the GO− group (15.7 ± 1.3 mm) (range 14.5–16.5 mm) (*p* < 0.001). The upper normal limit for children below 18 years old for our country was 15 mm [24]. Mild ocular motility impairment to the lateral side was seen in only one patient (5.6%). The severity of GO in most patients was mild, and no patients had corneal pathology, strabismus, or optic neuropathy.

The mean of clinical activity score was higher in the GO+ group (1.44 ± 1.28) (0–4) than in the GO− group (0.08 ± 0.27) (0-1) (*p* < 0.001). Most patients with GO+ had a mild activity of eye disease, and CAS more than 3 points was in 16.6% of GO+ patients. Eyelid redness and swelling were present in 33.3%, redness of caruncula lacrimalis and conjunctival injection were in 27.8% and 38.9%, respectively, and chemosis was present in 11.1% of GO+ patients.

An important finding was that the GD course in GO+ was longer than that in GO− patients. In GO− patients, euthyroidism was achieved within 16 months while in GO+ patients within 29 months (*p* < 0.001). The duration of the first remission was longer in GO− (13 months) than in patients with GO+ (5 months) (*p* < 0.001). Only one attack of GD within a follow-up period was noted in more patients with GO− (55.1%) than in GO+ (11.1%) (*p* = 0.001). The remission was not achieved within two years in 27.8% of GO+ patients and in 14.3% of GO− patients (*p* = 0.2). Relapses of hyperthyroidism in GO+ patients during one and two years after remission were more frequent in comparison with GO− patients. The duration of the first remission was longer than one year for 61.2% in GO− patients and 33.3% − in GO+ (*p* < 0.02).

Remission of Graves' hyperthyroidism was achieved after one (75.0%) or two years (62.5%) of discontinuation of antithyroid therapy for a lower percentage of GO+ smokers (active and passive) in comparison with nonsmokers (80.0% and 70.0%, resp.) (*p* > 0.05).

## 4. Discussion

GD and ophthalmopathy are uncommon in children. GO was present in 26.9% of our GD patients. Quite similar results were present in previous studies [1]. Other authors demonstrate that GO in juvenile GD occurs in 35–60% [5, 6, 8]. Our findings contrast with a study that reported that GO was found only in 17% of children with GD [7]. GO occurred together or to GD diagnosis in 83.3% of our patients. An explanation for simultaneous beginning of GD and GO might be the same autoimmune processes in the orbit and thyroid gland [6]. In our study, GD and ophthalmopathy were much more common in girls than in boys with a ratio 5 : 1. This is similar with other children GD studies [5–8]. Most of our patients had mild ocular findings consistent with GD. Common clinical manifestations in juvenile GO included upper eyelid retraction (72.28%), proptosis (66.7%), and soft tissue involvement (27.8–38.9%) with low CAS. Our results demonstrated that clinically active and severe GO was uncommon. Patients in our study had no corneal pathology, strabismus, or optic neuropathy. In another study, proptosis was present in 38%; eyelid retraction was noted in 23% of children with GD [7]. The palpebral fissure (12.9 ± 1.3 mm) in our GO+ patients was statistically significantly wider than that in GO− patients. Another study showed that lid aperture median was 10 mm (range 9–12 mm) in mild GO and 13 mm (10–17 mm) in moderate-to-severe GO [6]. Proptosis (17.4 ± 1.7 mm) was significantly greater in GO+ patients than in GO−. Normal exophthalmometric measurements in children and adolescents in our country were for 5–7 years children proptosis median was 13.2 mm, 8–11 years 14.9 mm, 12–15 years 15.5 mm, and 16–18 years 16.0 mm [24]. Other studies showed higher proptosis values: 18 mm in mild children GO and 22 mm in moderate to severe GO [6]. Studies showed that most of juvenile patients presented with mild proptosis, eyelid retraction, and soft tissue changes [5–7]. Researchers described that presenting signs of pediatric GO were milder than those of adults [1, 4, 5, 7, 8]. GD in children between 6 and 10 years of age is very rare (15%), and in teenagers, its incidence is about 80% [3]. Although antithyroid drug therapy is still the first-line treatment of Graves' hyperthyroidism in children in many European countries, radioactive iodine treatment in the USA is often used in children aged 10 years and older. Young children are less likely to achieve a remission on antithyroid drug treatment compared with adolescents, and long-term antithyroid drug therapy is therefore necessary [3]. There are no reports concerning the role of GO presentation on the course of juvenile GD despite the fact that the clinical course of the disease is dependent on a pediatric patients' age and the duration of antithyroid drug treatment. The literature data indicate that remission rates in children are less than 30% following two or more years of antithyroid drug therapy [3, 11, 16]. In some studies, remission rates after antithyroid drug treatment course were from 30% to 56% in children and adolescents with GD [2, 12, 13, 15]. However, Kim and Hwang [14] reported that only 12.2% of children with GD were in remission. Authors reported that prolonged therapy in children with GD was associated with increased remission rates [1, 3, 11, 16]. Younger children were found to be significantly associated with Graves' hyperthyroidism relapse. Authors reported that hyperthyroidism relapse rates for children were 59% one year and 68% two years after the course of antithyroid drug treatment. Relapse risk decreased with longer duration of the first course of therapy. Relapse rate 2 years after the end of treatment was 83% in patients treated for less than two years and 60% in patients treated for more than two years [16]. Our GD patients with GO were treated more than two years (29 months), but the relapse within one year after remission was in 50.0% of patients with GO and within two years 57%. On the basis of our findings, patients with GO had more frequent hyperthyroidism relapses. In our GD patients without GO, relapse rate was 39% within 1 year and 37% within 2 years after remission. Other authors show that 34% to 42% children with GD experienced relapse [2, 13]. The risk of relapse decreased with increasing age of children at the onset of disease and duration of the first course of antithyroid drug therapy [16]. The follow-up of our patients with GD revealed that patients with accompanying GO had longer time to achieve permanent euthyroidism, shorter duration of remission, and more frequent relapses.

We have not found publications which include data regarding the impact of GO and smoking on the influence on juvenile GD course. Our study demonstrated the influence of smoking on GD course. Remission of Graves' hyperthyroidism was achieved after one or two years of discontinuation of antithyroid therapy for a lower percentage of GO+ smokers (active and passive) in comparison with nonsmokers. However, the number of smokers was too small to draw well-founded conclusions regarding the association between smoking and GD course. Authors report that the prevalence of smoking is much lower in children 4% than in adults 47% [1, 25]. Smoking is a modifiable risk factor for GO and significantly increases the risk of development and the severity of GO. Active cigarette smoking is unusual in childhood. Our active smoking patients were teenagers of 15–18 years old. The prevalence of smoking among adolescents is related to the prevalence of juvenile GO and GD. Interestingly, pediatric GO and GD manifestations are more similar to that of an adult [1, 3]. This can be explained by increasing prevalence of smoking with age as smoking is proven as a risk factor for GO and GD development [26]. Passive smoking may also have a detrimental effect on the pediatric GD course and increase GO progression [26]. A recent study confirms these findings and suggests that the progression and severity of GD also depend on exposure to tobacco smoke.

Several studies in adults have tried to identify factors which are associated with the recurrence of GD: young age [16, 20, 27], smoking [10, 17, 25, 28], and the presence of GO [9, 19, 20]. Our study results support the more severe course of autoimmune thyroid disease in patients with GO. While our study findings are in concordance with some previous adult studies [9, 18–20], there are also conflicts among the results of other authors [10, 17, 21, 29, 30]. Authors showed that nonsmokers and patients with GD and without ophthalmopathy had a longer duration of remission [9, 18]. Despite the absence of statistical relations between our patients, smoking (active and passive) should still be regarded as harmful to children and adolescents with GD and ophthalmopathy. Our study suggests that cessation of smoking (active or passive) can reduce the progression of GD course and improve the response to treatment. Avoiding passive smoking in children with GD can be beneficial. Tobacco exposure and smoking household members should also be avoided as well. Stopping smoking and euthyroidism restoring, until the eye disease is inactive, can prevent GO from progressing.

The current study is the first one in Lithuania and the largest retrospective study done in juvenile patients with GD regarding the GD course and the presence of GO. Our findings confirm that GO is one of the factors in determining the long-term outcomes of GD (remission and recurrence). The closer interaction in daily practice between endocrinologists, family doctors, and ophthalmologists could play a major role in thyroid and ocular signs in GD and treatment outcomes. The study is helpful to improve the prognosis of GD and understand which patients are more or less likely to experience GD relapse. We recommend close monitoring for GD recurrences in patients with GO.

Our investigations of patients with juvenile GD suggest the requirement for further prospective study in order to develop a diagnostic and treatment algorithm and to improve the outcomes of GD.

## 5. Conclusion

The common manifestations of juvenile GO patients were upper eyelid retraction, proptosis, and soft tissue involvement. The study demonstrates that pediatric patients with GO are more likely to have a severe course of autoimmune thyroid disease: the longer the course of GD, the more frequent the relapses and shorter duration of remission.

## Figures and Tables

**Table 1:** Characteristics of patients with GD.

Characteristics	Median (25–75 percentiles)			*p*
	All sample (*n* = 67)	GO− (*n* = 49)	GO+ (*n* = 18)	
Age at diagnosis, year	13.3 ± 2.3	12.7 ± 2.9	13.9 ± 3.4	0.09
Active smoking, *n* (%) Passive smoking, *n* (%)	9 (13.4) 14 (20.1)	6 (12.2) 9 (18.4)	3 (16.7) 5 (27.8)	0.6 0.4
Proptosis, mm	16.6 ± 1.2	15.7 ± 1.3	17.4 ± 1.7	<0.001
Palpebral fissure, mm	11.9 ± 0.9	11.3 ± 0.6	12.9 ± 1.3	<0.001
CAS	0.45 ± 0.8	0.08 ± 0.27	1.44 ± 1.28	<0.001
Duration of the first treatment, months (range)	19 (12–34)	16 (12–28.5)	29 (17.5–34)	<0.001
Duration of the first remission, months (range)	10.2 (2–22.5)	13 (5–22.5)	5 (2–11.5)	<0.001
There was noted only one attack of GD, *n* (%)	31.8 (28)	55.1 (27)	11.1 (2)	0.001
Relapse within 1 year after remission, *n* (%)	41.8 (28)	38.8 (19)	50.0 (9)	0.4
Relapse within 2 years after remission, *n* (%)	38.8 (26)	36.7 (18)	56.6 (10)	0.2
Duration of the first remission more than 1 year, *n* (%)	58.2 (39)	61.2 (30)	33.3 (6)	0.02
GD recurrence within 2 years post therapy, *n* (%)	16.4 (11)	14.3 (7)	27.8 (5)	0.2
